# Supplementary material for: Formulated Phospholipids as Non-Canonical TLR4 Agonists
Source: Pharmaceutics. 2022 Nov 22;14(12):2557. doi: 10.3390/pharmaceutics14122557 (PMC9788208; doi:10.3390/pharmaceutics14122557)
Supplement: Supplementary file 1 [file pharmaceutics-14-02557-s001.zip › pharmaceutics-2029788-supplementary.pdf]

Supplementary Materials

Supplementary Table S1. Liposomal particle size and polydispersity index (PDI). All measurements are shown as the mean of 3 replicate measurements ± 1 SD.

Liposome Composition	Z-Average Particle Size (nm.d)	PDI
DLPC	57.9±0.54	0.288±0.030
DMPC (Batch 1)	71.6±0.87	0.267±0.001
DMPC (Batch 2)	71.7±0.44	0.257±0.003
DPPC	54.2±0.63	0.181±0.003
DSPC	56.2±0.18	0.160±0.005
DOPC	75.9±0.89	0.203±0.008
POPC	85.5±0.15	0.221±0.007
DMPG	57.2±0.71	0.287±0.17
DMPS	52.4±0.19	0.152±0.002

Supplementary Table S2. Emulsion and other formulation particle size and polydispersity index (PDI). All measurements are shown as the mean of 3-9 replicate measurements ± 1 SD.

Emulsion Composition	Z-Average Particle Size (nm.d)	PDI
Squalene SE (Batch 1)	95.7±1.22	0.044±0.009
Squalene SE (Batch 2)	106.9±2.67	0.042±0.031
Squalene SE (Batch 3)	90.9±1.43	0.043±0.018
Grapeseed SE	89.5±2.24	0.083±0.025
Grapeseed SE (Batch 2)	95.2±1.69	0.068±0.019
GLA-AF	41.3±1.18	0.200±0.002
Squalene MF59-Like (Batch 1)	153.1±2.37	0.106±0.015
Squalene MF59-Like (Batch 2)	143.9±3.74	0.026±0.016
Grapeseed MF59-Like	150.5±4.02	0.035±0.019
